# Assessment of Knowledge, Attitude, and Disposal Practice of Unused and Expired Pharmaceuticals in Community of Adigrat City, Northern Ethiopia

**DOI:** 10.1155/2020/6725423

**Published:** 2020-04-14

**Authors:** Halefom Kahsay, Mubarek Ahmedin, Binyam Kebede, Kiflay Gebrezihar, Haylay Araya, Desta Tesfay

**Affiliations:** ^1^Department of Pharmacy, College of Medicine and Health Science, Adigrat University, Adigrat, Ethiopia; ^2^Independent Researcher, Dire Dawa, Ethiopia; ^3^Department of Pharmacy, College of Health Science, Debre Markos University, Debre Markos, Ethiopia; ^4^Department of Pharmaceutics, School of Pharmacy, College of Health Science, Mekelle University, Mekelle, Ethiopia

## Abstract

**Background:**

Medicines have become part of our day-to-day life. Due to different reasons, patients may not use all the medications dispensed to them. The storage of drugs at home promotes self-medication, which results in variety of adverse consequences. Global growth in health-seeking awareness and behavior among people has resulted in increment of medicine consumption over years. However, Ethiopians have little awareness about proper disposal of unused and expired pharmaceuticals. Besides, large quantities remain unused or expired since not all medications given to the consumers are consumed. Hence, this study could serve as an indicator for the country policy makers concerning pharmaceutical waste management.

**Objective:**

To assess knowledge, attitude, and disposal practice of unused and expired pharmaceuticals in the community of Adigrat city, Tigray, Ethiopia, 2019. *Methodology*. A cross-sectional study was conducted among 359 respondents from the residents of Adigrat city. Semistructured questionnaires, which focused on knowledge, attitudes, and disposal practices for unused and expired medications, were used to collect data from respondents. Epi-data 3.0 suite and the statistical package for social sciences (SPSS) version 20 were used in data entry and analysis.

**Results:**

All of the 359 returned questionnaires were valid for data entry and analysis. Of the 359 respondents, 57.7% were men and the majority (93%) were Orthodox Christians. Almost half of the respondents (50.14%) have good knowledge concerning the disposal of unused and expired pharmaceuticals. Most (82.2%) of the respondents have a positive attitude towards the disposal of unused and expired pharmaceuticals. Around fifty-two (52.4) of the respondents had unused medicines stored at home, with analgesics being the most common (41.5%). Around three-quarters (75.2%) and 63% of the respondents discarded unused and expired medicines in the garbage bins, respectively.

**Conclusion:**

Although the majority of the respondents had a positive attitude towards the disposal of unused and expired medications, almost half of the sample population were unaware of proper disposal practices. Furthermore, less were inclined to practice proper disposal of unused and expired medications in the city. Therefore, we recommend further studies that focus on how the disposal attitude of the population can influence their knowledge and practice of the disposal of unused and expired medications.

## 1. Background

Medicines have become part of our day-to-day life. Global growth in health-seeking awareness and behavior among people has resulted in increased consumption of medicines over the years. However, patients may not use all of the medications dispensed to them because of many reasons. These include relief of the symptoms, forgetfulness, dosage changes, side effect intolerance, and medications reaching the expiration date. As per the WHO, 50% of medicines are prescribed, dispensed, or sold inappropriately, and half of all patients fail to take them correctly. More than half of all medications are inappropriately prescribed and sold, which poses a risk to environment [1–4]. It can also impose a huge economic burden on the patients as well as the healthcare system. It has been also estimated that billions of dollars' worth of unused medication is wasted every year [5, 6]. The storage of drugs at home promotes self-medication, which results in a variety of adverse consequences [7–10]. For example, it leads to incidences of drug resistance, loss of potency, toxicity risk, environmental pollution, and incomplete dosage. Although there are options for disposing of unused drugs, many consumers keep drugs in their possession due to unawareness of how to dispose of them properly. In addition, a recent review suggests that consumers use different methods for disposing unused medicines, with the most commonly being throwing medicines in garbage, toilet, or sink [11, 12].

Although a few studies have been done to assess the knowledge, attitude, and disposal practice of unused and expired pharmaceuticals among households in Ethiopia, there is still a dearth of information regarding the disposal practices for unused medications in areas like Adigrat city. Therefore, this study was conducted to assess the knowledge, attitudes, and disposal practices for unused and expired medications among the residents of Adigrat city. Generally, this survey could serve as an indicator for environmental policy makers. On the other hand, it could be used as indicator for responsible offices of Adigrat city to take appropriate measures regarding the disposal of unused and expired pharmaceuticals.

## 2. Methods

**Figure 1:**
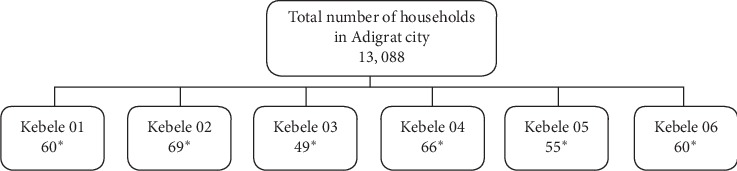
The schematic presentation of total number of households and sample taken in each kebele of Adigrat city, 2019.

A community-based cross-sectional study with a multistage-sampling technique was conducted in Adigrat city, Tigray regional state, Northern Ethiopia. Individuals over 18 years (207 males and 152 females) with sound mental health were selected for the study. The sample size was calculated using a single population proportion formula, by using a margin of error of 0.05 and a proportion of event occurrence at 66.6% similar to another study conducted in Eastern Ethiopia at a 95% of level of significance by adding a 5% nonresponse rate. Based on the above assumption, the minimum sample size required for the study was 359. Proportional random sampling technique was used to select households from each kebele (i.e., the smallest administrative unit of Ethiopia) of the Adigrat city (Figure 1). Data was collected through interviews using a previously adopted standard questionnaire that was also translated into local languages. The translated data was then checked for errors in communication resulting from mistranslations. Data analysis was performed using SPSS Statistics software. Sociodemographic data and the levels of KAP of the study participants related to unused and expired pharmaceuticals were measured, with frequencies for descriptive variables and binary logistic regression undertaken, to explore the factors associated with the knowledge, attitudes, and practices of the respondents (*P* ≤ 0.05) at a 95% confidence interval (CI).

Bivariate association between dependent and independent variables was determined, and covariates with *P* ≤ 0.2 with the outcome variable were dropped from further consideration in modeling by using multivariate analysis as an adjusted OR (AOR). Individuals who had a knowledge score of at least three out of the five questions were considered as having good knowledge concerning unused and expired medication disposal, and those who scored less than two were considered as having poor knowledge [13]. Similarly, those individuals who answered with “strongly agree” and “agree” to the questions were considered to have a positive attitude concerning unused and expired medication disposal, while those who answered with “strongly disagree” and “disagree” to the questions were considered to have a negative attitude. The study was reviewed by the Ethics Committee of the School of Pharmacy at Adigrat University and was granted ethical clearance. Permission was also obtained from the Adigrat city Health Office to conduct the study in the city. Prior to data collection, informed consent was obtained from the participants.

## 3. Results

### 3.1. Sociodemographic Characteristics of the Respondents

All the approached 359 individuals agreed to participate in the study. Of the 359 respondents, 207 (57.7%) were males. The majority (137; 38.2%) of the respondents were 32 years old and above. Concerning their educational level, one hundred and twelve (31.2%) respondents completed secondary education, 178 (49.6%) had a college/university degree and above, and 31 (8.6%) were illiterate. Of the total respondents, 334 (93%) were Orthodox Christians (Table 1).

### 3.2. Respondents Knowledge concerning Unused and Expired Medication Disposal

**Figure 2:**
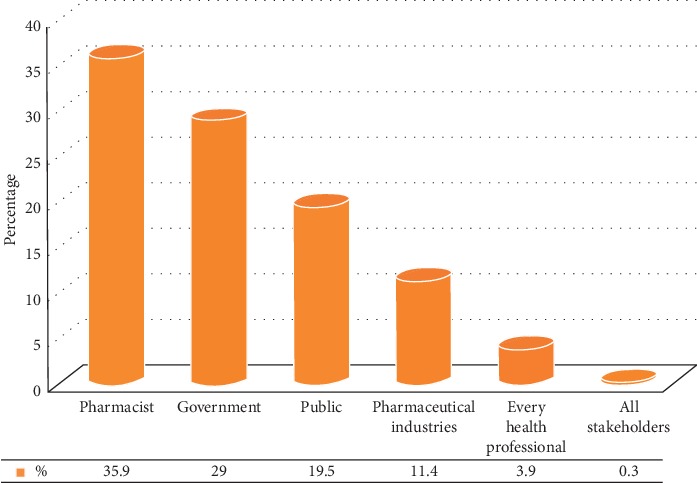
Bodies responsible for creation of awareness about proper medicine disposal in Adigrat city, Northern Ethiopia, 2019 (*N* = 359).

As presented in Table 2 the respondents gave possible answers about awareness of unused and expired medication disposal. On assessing the knowledge score, this study found that about 50.14% of respondents have good knowledge. Furthermore, most of the respondents (87.2%) knew about drug resistance. On the other hand, a large portion of the respondents (87.2%) did not know about drug-take-back systems. The majority of the respondents (95%) felt that improper disposal of unused and expired medicines has detrimental effects on the environment and health. In multivariate logistic regression tests, those who did not have any level of educational attainment were nearly 0.66 times (AOR = 0.338, 95% CI = 0.102–1.121) more likely to have poor knowledge concerning unused and expired medication disposal in their households compared to those who have college/university and above educational status. However, the presence of higher education levels attained in households did not show a statistically significant difference in unused and expired medication disposal (Table 3). A majority (35.9%) of the respondents responded as pharmacists were the responsible bodies in creation of awareness about proper disposal of medicines to them (Figure 2).

### 3.3. Respondents Attitude concerning Unused and Expired Medication Disposal

**Figure 3:**
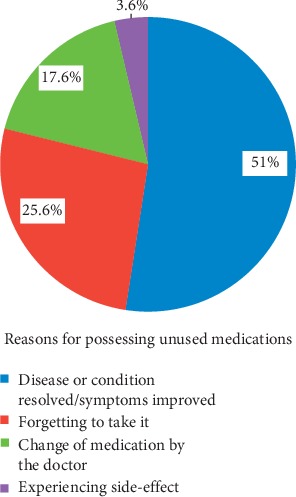
Respondents' reasons for keeping purchased medicine unused at home in Adigrat city, Northern Ethiopia, 2019 (*N* = 359).

**Figure 4:**
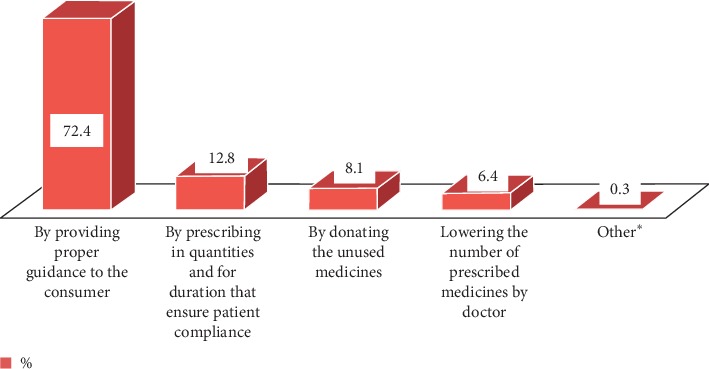
Actions that should be taken to minimize or control the hazardous effect of unused and expired medicines in Adigrat city, Northern Ethiopia, 2019 (*N* = 359). ^*∗*^Keeping in safe place, disposing in toilet, and burning.

**Figure 5:**
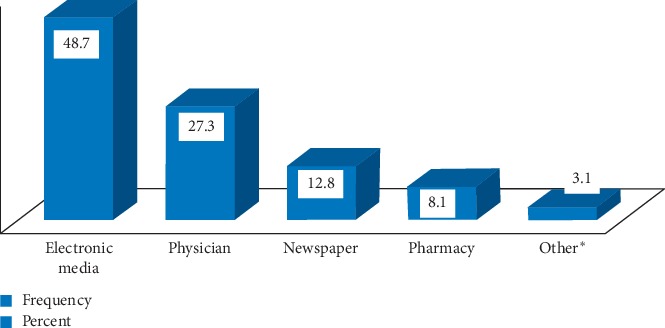
Householders' attitudes towards best source of awareness for society in Adigrat city, Northern Ethiopia, 2019 (*N* = 359). ^*∗*^Health bureau, environmental agency, and drug regulatory agency.

Possession of unused medication was mostly due to resolving disease or conditions/improving symptoms (51%), forgetting to take it (25.6%), changing of medication by the doctor (17%), and experiencing side-effects (3.6%) (Figure 3). As shown in Figure 4, most of the respondents (72.4%) suggested that proper guidance should be given to the consumer. In response to a question about the best means to create awareness of proper drug disposal practices, 48.7% considered advertising through electronic media and 27.3% considered the physician responsible, while the rest were in favor of all sources, including the pharmacy staff and newspapers (Figure 5).

On assessing attitude scores, over eighty-percent (82.2%) of the respondents have positive attitude towards the disposal of unused and expired pharmaceuticals. Nearly half (48.7%) of the respondents “strongly agreed” to the extent that they believe that unused and expired medicines present potential risks or negative consequences at home. Additionally, 44.8% of the respondents stated that they “strongly agreed” that the risks of unused and expired medicines to children are greater, while 24.5% of the respondents “strongly agreed” that a lack of adequate information on safe disposal practices is a precursor to the risks and negative consequences of unused and expired medicines. In contrast, 1.9% of the respondents “strongly disagreed” that there is adequate advice by doctors and healthcare professionals on safe disposal practices with a further 2.5% “strongly disagreeing” that a lack of adequate information on safe disposal practices is a precursor to the risks and negative consequences of unused and expired medicines. Concerning take-back programs, the respondents gave varying opinions. Over ten percent (11.4%) strongly agreed that the programs should be mandatory. However, the respondents inclined to accept mandatory take-back programs (25.9%) were fewer than those who were not in favor of them (62.1%), whereas only 12% remained neutral (Table 4).

### 3.4. Respondents Practice on Unused and Expired Medication Disposal

**Figure 6:**
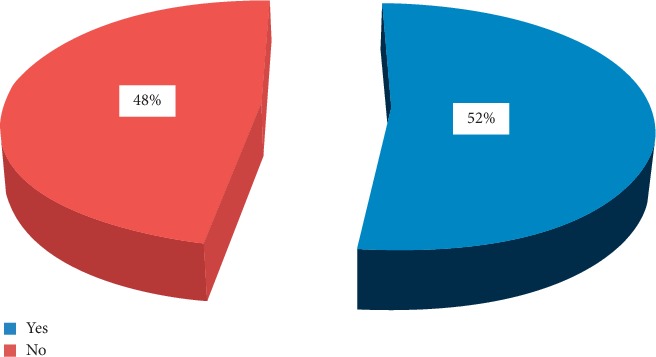
Purchased medicines remaining unused at home among households in Adigrat city, Northern Ethiopia, 2019 (*N* = 359).

Approximately 52.4% of the respondents had unused medicine stored at home, but 47.6% of the respondents did not have unused medicines stored at home **(**Figure 6**).**

The common types of medicines kept in households were analgesics (41.5%) and antibiotics (36.7%). In addition, antipain and antibiotic (4.8%), antidiabetic (5.3%), and antihypertensive (8%) medicines were other types of unused medications found in homes (Table 5).

The most preferred disposal practice for unused medicines was throwing them in garbage bins (63%) followed by giving them to friends or relatives 13.9%. Some of respondents (4.2%) keep unused medicines at home until they become expired, while 1.9% and 6.7% of the respondents indicated that they burn and donate medicines to hospitals, respectively. About (7%) respondents considered that returning unused medicines to the pharmacies would be the best option (Table 6).

The most preferred disposal practice for expired medicines was throwing them in garbage bins (75.2%) followed by flushing them in the toilets (15%). Some respondents (3.9%) said that they did not know what to do with the expired medicines, while 2.5% and 0.8% of the respondents reported that they would return them to pharmacy and give them to friends or relatives, respectively (Table 7).

Above two-third of respondents discarded the expired medications as they are, 5.8% did not know about the practice of expired medication disposal, 8.1% crushed them before discarding, and only 4.2% said that expired medications are thrown after dilution (Table 8).

## 4. Discussion

The proper collection and disposal of unused and expired medications through a well-run disposal system and collection programs have implications for ensuring public safety for humans and safeguarding the natural environment. Although medication disposal is a hot topic gaining attention across the world because it has been realized that improper disposal can contaminate the environment [14, 15], this study shows that over eighty percent (87.2%) of the respondents did not know about drug-take-back systems, which is consistent with studies conducted in Bangladesh (96.8%) and Malaysia (93.6%) [6, 16] and higher than a study conducted in Harar city (66.9%) [17]. Even though 12.8% of the respondents in the current study knew about drug-take-back systems, only 7% and 2.5% of the respondents returned unused and expired medicines back to the pharmacy, respectively. This might be due to a lack of established drug-take-back system in Adigrat city. On the other hand, most of the respondents (87.2%) knew about drug resistance; although this was lower than a study conducted in Bangladesh (96.55%) [6], it is higher than that in Harar city (78.1%) [17]. The difference from the study conducted in Harar city might be because pharmacists and other healthcare professionals in Adigrat city create adequate awareness about drug resistance and its associated problems. The emergence of resistance during therapy has been shown to significantly affect outcomes negatively [18].

The majority of the respondents (95.8%) were aware of the hazardous environmental and health impact of improper disposal of unused and expired medicines. This is comparable to findings in Afghanistan, where most participants (98%) felt that improper disposal of unused and expired medicines can affect the environment and health [19]. However, this finding is higher than the results from Karachi city (80%) and Harar city (86%) [17, 20]. This study found that about 50.14% of the respondents have good knowledge. This might be because healthcare professionals in Adigrat city show their interest in creating awareness about the negative impacts of improper disposal of unused and expired medicines. It may also be because governmental bodies provide adequate training for those healthcare professionals about this issue [1].

The current study shows that 52.4% of the respondents had leftover, unused, or unwanted medications, which is lower than the results of studies conducted in India and Harar city, where 68 and 66% of the respondents, respectively, stored unused medicines at home [17, 21]. In this study, the common types of medications kept in households were analgesics (41.5%) and antibiotics (36.7%), which is inconsistent with studies conducted in Harar, USA, and Nigeria for analgesics (62.7%, 15%, 18.6%) and antibiotics (24%, 6.7%,16.8%), respectively [5, 17, 22]. An excess of those medications at home may be due to the idea of ready prevention of infectious diseases without referral to a doctor, and pain is associated with many diseases that patients may consider treatable with analgesics in Adigrat city. Therefore, these results imply a high prevalence of self-medication. Indeed, an increased amount of antibiotics at home may lead to antibiotic resistance. The storage of unwanted or unused medication in the household provides an increased risk of accidental childhood poisonings. Reasons for possessing unused medication were mostly a disease or condition that resolved, with symptoms improved (51%), or forgetting to take it (25.6%). This excess of medications in homes leads to the issue of inappropriate disposal and has potential implications for accidental childhood ingestion [23, 24].

In order to minimize the entry of pharmaceuticals into environment, 72.4% of the respondents in this study suggested that proper guidance should be given to the consumer and 12.8% of the respondents suggested that medication should be prescribed in smaller quantities and only for time periods that ensure patient compliance. This result is inconsistent with a study conducted in Karachi city where 33% suggested that proper guidance should be given to the consumer, with 37% of the respondents advocating for short-term trial prescriptions [6]. In contrast, 68.6% of the respondents advocated for more guidance that is proper and 17.7% for trial prescriptions in the Harar study [17]. Differences in suggestion might be affected by the inadequate level of advice given by health professionals in Adigrat. Because this study found that about 82.2% of the respondents have positive attitude towards disposal of unused and expired pharmaceuticals, it is likely that better advice and guidance from healthcare professionals may contribute to a better practice of proper disposal.

The most preferred disposal practice for unused medicines was throwing them in garbage bins (63%) followed by giving them to friends or relatives (13.9%), while some respondents suggested donation to pharmacy (7%). From a study carried out in New Zealand, it became evident that more than 35% of the respondents considered it acceptable to flush down unused medicines in the toilet and more than 21% believed that it is acceptable to rinse them down the kitchen sink [19]. Another study conducted in Harar city showed that most of the respondents preferred throwing unused medications in garbage bins (53.2%) followed by flushing them in the toilets (23.9%), while some respondents (16%) kept unused medicine at home until they expired [17]. This difference might be due to garbage bins being more easily available around compounds and lack of awareness about proper disposal practices of medications in Adigrat city

Approximately two-thirds (75.2%) of respondents in this study assert that they throw away the expired medicines in the household trash while 15% of the respondents flush down expired medicines in the toilets. From a study carried out in Afghanistan, it is evident that around 78% of the respondents throw away expired medicines in household garbage and 12% of respondents flush expired medications in toilet or sink [19]. A study conducted in Harar shows that one-half (50%) of respondents throw away the expired medicines in the household trash while 37.2% of the respondents flush down expired medicines in the toilets. This difference might be because respondents believe that expired medications should be flushed down into the toilet in order to prevent accidental childhood and environmental poisoning. According to the current study, most of the methods for disposal of unused and expired medicines followed by the respondents are not recommended methods, though they are commonly used in many places. Nevertheless, the recommended method for disposal of most pharmaceuticals is high temperature incineration (as it oxidizes all organic material into carbon dioxide and renders them inert to any toxicological effect in the environment), which requires some initial organized method of collecting and sorting. Nowadays, in many developed countries, drug-take-back systems are established for collection of unused and expired medicines. One type of drug-take-back program that has been suggested by “Nebraska Medication Education for Disposal Strategies (MEDS)” is to put tamper-resistant boxes in pharmacies that will allow consumers to bring medicines back to knowledgeable pharmacists. For example, in Sweden and Korea, more people return unused medicines to a pharmacy for correct disposal, as they have realized the environmental concerns posed by expired medicines [25–27].

In order educate the community about proper disposal of unused and expired medication, it is important to increase awareness among the public through the government, pharmacists, and pharmaceutical industries. A significant role can be played by community pharmacists being on the forefront in guiding the community and providing it with proper education and awareness [28]. Therefore, the government of Ethiopia should be committed to encourage feasible expired pharmaceutical collection programs. However, stronger campaigns and significant involvement of the patient, healthcare professionals, and the government officials are required, in order to avoid any possible barriers such as lack of information about techniques of proper disposal of expired medication [29]. Additionally, the White House Office of National Drug Control Policy recommends household trash disposal if take-back programs are unavailable. Prescription medicines along with the patient information should be removed from their original containers, mixed with some undesirable substance, such as kitty litter, coffee grounds, or sawdust inside a sealable plastic bag container, and disposed of in the trash. In addition, the American Pharmacists Association also recommends that unwanted medications should be crushed or dissolved in water prior to mixing with the undesirable substance [30, 31].

Above two-thirds (81.9%) of respondents in the current study discarded expired medications. However, the FDA recommends that expired medications should be initially mixed (without crushing tablets or capsules) with an unpalatable substance such as dirt, cat litter, or used coffee grounds. Then, the mixture should be placed in a container such as a sealed plastic bag, and the container should be thrown in household trash. However, before throwing the container, all personal information on the prescription label of empty pill bottles or medicine packaging should be deleted [32]. Multivariate logistic regression tests showed that sociodemographic characteristics of the respondents affected knowledge concerning unused and expired medication disposal. Those with no formal educational achievements were nearly 0.66 times (AOR = 0.338, 95% CI = 0.102–1.121) more likely to have poor knowledge concerning unused and expired medication disposal in their households compared to those who have college/university and above education status. However, the presence of highest education levels attained in households did not show a statistically significant difference in knowledge from those with lower standards. On the other hand, those who are Orthodox Christians are nearly 11 times more likely to have knowledge about unused and expired medication disposal in their households compared with those who followed Catholicism (AOR = 10.797, 95% CI = 1.205–96.717). However, this result is tentative since disparities in the sampling between participants from different religions could exist. Therefore, further investigations are needed to justify this finding. Indeed, the limitations of the study, most importantly the cross-sectional nature of the study design, prevent us from drawing causal inferences about the relationship between the chosen covariates and outcome variables over a period. Based on the findings, it could be a challenge to infer the present results about the status of knowledge, attitudes, and disposal practices of unused and expired pharmaceuticals in other areas of Ethiopia. Additionally, this study has a dispute in data collection because of the dislocation existence of new households and old data taken regarding the houses included in the study area.

## 5. Conclusion

The study indicates that improper discarding of medications seems to be practiced in Adigrat city. A large portion of the respondents did not know about drug-take-back systems. On the other hand, most of the respondents had a positive attitude towards the risks of unused and expired medications. Almost half of the respondents suggested that awareness regarding disposal of unused and expired pharmaceuticals should be improved. There is a pressing need for increasing awareness about proper disposal of unused and expired medicines among the public. Community pharmacists can play a significant role in encouraging proper disposal practices in Adigrat city.

## Figures and Tables

**Table 1:** Sociodemographic characteristics of respondents in Adigrat city, Northern Ethiopia, 2019 (*N* = 359).

Variables and categories	(*N* = *359)*	%
*Gender*		
Male	207	57.7
Female	152	42.3
*Age*		
18–24	134	37.3
25–31	88	24.5
>31	137	38.2
*Marital status*		
Single	134	37.3
Married	207	57.7
Divorced	6	1.7
Widowed	12	3.3
*Profession/occupation*		
Governmental employee	108	30.1
Self-employed	133	37
Housewife	29	8.1
Farmer	8	2.2
Student	79	22
Other^*∗∗∗*^	2	0.6
*Monthly income (ETB) * ^*∗*^		
<1380 (poor)	156	43.5
1381–6900 (low)	155	43.2
6901–13800 (middle)	45	12.5
>13800 (high)	3	0.8
*Education level*		
Illiterate	31	8.6
Primary [1–8]	38	10.6
Secondary [9–12]	112	31.2
College/university student and	178	49.6
Above		
*Religion*		
Orthodox	334	93
Muslim	12	3.3
Protestant	2	0.6
Catholic	11	3.1

^*∗*^Classified according to World Bank income level scale for developing countries; ETB: Ethiopian birr; ^*∗∗∗*^unemployed.

**Table 2:** Respondents knowledge concerning unused and expired medication disposal in Adigrat city, Northern Ethiopia, 2019 (*N* = 359).

No.	Questions	Answer	
		**Yes**	**No**
1	Do you know about medication waste?	178 (49.6%)	181 (50.4%)

2	Have you ever read medicines disposal instructions?	143 (39.8%)	216 (60.2%)

3	Do you know about “drug-take-back system”?	46 (12.8%)	313 (87.2%)

4	Do you know that misused/repeated change or not complete antibiotics may cause drug resistance?	313 (87.2%)	46 (12.8%)

5	Do you know that improper disposal of unused and expired medicines can affect the environment and health?	344 (95.8%)	15 (4.2%)

	^*∗*^Knowledge score		
	•Good knowledge: 180 (50.14%)		
	•Poor knowledge: 179 (49.86%)		

^*∗*^ Score 0–2: poor knowledge, score 3–5: good knowledge.

**Table 3:** Multivariate logistic regression analysis results of sociodemographic characteristics of the respondents associated with their knowledge about the disposal of unused and expired medication in Adigrat city, Northern Ethiopia, 2019.

Characteristics		COR		AOR	
		COR (CI95%)	*P*-value	AOR (CI 95%)	*P*-value
Sex	Male	1.010 (0.65–1.535)	0.964	0.825 (0.499–1.366)	0.456
	Female	1.00		1.00	
Age (in years)	18–24	2.016 (1.243–3.270)	**0.004**	1.824 (0.875–3.798)	0.109
	25–31	1.404 (0.819–2.405)	0.217	1.506 (0.800–2.833)	0.204
	>31	1.00		1.00	
Marital status	Single	0.741 (0.212–2.583)	0.638	0.767 (0.189–3.111)	0.711
	Married	0.377 (0.110–1.292)	0.121	0.528 (0.134–2.083)	0.361
	Divorced	0.500 (0.068–3.696)	0.497	0.826 (0.087–7.856)	0.868
	Widowed	1.00		1.00	
Profession/occupation	Governmental Employee	0.964 (0.059–15.805)	0.979	0.980 (0.051–18.962)	0.990
	Self-employed	0.928 (0.057–15.141)	0.958	1.226 (0.065–23.011)	0.892
	Housewife	0.611 (0.035–10.794)	0.737	1.448 (0.067–31.230)	0.813
	Farmer	1.667 (0.074–37.728)	0.748	10.024 (0.299-336.469)	0.199
	Student	1.394 (0.084–23.099)	0.817	2.388 (0.118–48.405)	0.571
	Other	1.00		1.00	
Monthly income (in ETB birr)	≤ 1380	0.429 (0.038–4.825)	0.493	0.174 (0.013–2.362)	0.189
	1381–6900	0.457 (0.041–5.142)	0.526	0.387 (0.031–4.850)	0.462
	6901–13800	1.231 (0.103–14.778)	0.870	1.262 (0.099–16.233)	0.856
	>13800	1.00		1.00	
Education	Illiterate	0.253 (0.107–0.597)	**0.002**	0.338 (0.102–1.121)	0.076
Level	Primary school [1–8]	0.379 (0.182–0.788)	**0.009**	0.581 (0.235–1.433)	0.238
	Secondary school (9-12)	0.728 (0.453–1.171)	0.191	0.892 (0.497–1.600)	0.700
	College/university and above	1.00		1.00	
Religion	Orthodox	11.139 (1.410–87.996)	**0.022**	10.797 (1.205–96.717)	**0.033**
	Muslim	3.333 (0.292–38.082)	0.333	3.599 (0.266–48.641)	0.335
	Protestant	0.000	0.999	0.000	1.000
	Catholic	1.00		1.00	

The relationship is significant at *p* < 0.05.

**Table 4:** Attitudes towards unused and expired medicines among households in Adigrat city, Northern Ethiopia, 2019 (*N* = 359).

No.	Attitudes of respondents	Number of respondents (%)				
		Strongly disagree	Disagree	Neutral	Agree	Strongly agree
1	Unused and expired medicines present potential risks at home	4 (1.1)	6 (1.7)	4 (1.1)	170 (47.4)	175 (48.7)

2	Children are more vulnerable	3 (0.8)	4 (1.1)	1 (0.3)	190 (52.9)	161 (44.8)

3	Lack of adequate information on	9 (2.5)	13 (3.6)	12 (3.3)	237 (66)	88 (24.5)
	safe disposal					

4	Advise by doctors and healthcare professionals should be given	7 (1.9)	24 (6.7)	11 (3.1)	207 (57.7)	110 (30.6)

5	Take-back programs of unused and expired medicines should be mandatory	78 (21.7)	145 (40.4)	43 (12)	52 (14.5)	41 (11.4)

	Specific response	7	14	43	214	81
	^*∗*^Attitude status score					
	•Positive attitude: 295 (82.2%)					
	•Negative attitude: 21 (5.8%)					

^*∗*^Score for positive attitude: “strongly agree” and “agree”; score for negative attitude: “strongly disagree” and “disagree.”

**Table 5:** Types of medications remaining unused at home among households in Adigrat city, Northern Ethiopia, 2019 (*N* = **359)**.

Types of medications remaining unused at home	*N*	**%**
Analgesic	78	41.5
Antibiotic	69	36.7
Antihypertensive	15	8
Antidiabetic	10	5.3
Both analgesic and antibiotic	9	4.8
Other medications^*∗*^	7	3.7

^*∗*^Antihelminthics.

**Table 6:** Disposal practice of unused medicines among households in Adigrat city, Northern Ethiopia, 2019 (*N* = 359).

Disposal practice of unused medicines	*N*	%
Throwing them away in household garbage	226	63
Donating them to hospital	24	6.7
Giving them to friends or relatives	50	13.9
Returning them back to pharmacy	25	7
Keeping them at home until expired	15	4.2
Flushing them in toilet	12	3.3
Burning them	7	1.9
Total	359	100

**Table 7:** Disposal practice of expired medicines among households in Adigrat city, Northern Ethiopia, 2019 (*N* = 359).

Disposal practice of expired medicines	*N*	%
Throwing them away in household garbage	270	75.2
Flushing them in toilet	54	15
Giving them to friends or relatives	3	.8
Returning them back to pharmacy	9	2.5
Do not know	14	3.9
Other	9	2.5
Total	359	100

**Table 8:** Ways of discarding expired medicines among households in Adigrat city, Northern Ethiopia, 2019 (*N* = 359).

Ways of discarding expired medicines	*N*	%
Crushing them before discarding	29	8.1
Diluting them with water	15	4.2
As they are	294	81.9
Do not know	21	5.8
Total	359	100

## Data Availability

The dataset used to support the findings of this study are available from the corresponding author upon request.

## Abbreviations

APHA: American Pharmacists Association
CSA: Central statistical agency
FDA: Food and Drug Administration
HH: Households
NSAID: Nonsteroidal anti-inflammatory drug
OTC: Over the counter
ONDCP: Office of National Drug Control Policy
UEM: Unused and expired medicines
FMHACA: Food, Medicine and Healthcare Administration and Control Authority.
